# The Perceived Size and Shape of Objects in Peripheral Vision

**DOI:** 10.1177/2041669516661900

**Published:** 2016-08-17

**Authors:** Joseph Baldwin, Alistair Burleigh, Robert Pepperell, Nicole Ruta

**Affiliations:** School of Art & Design, Cardiff Metropolitan University, Cardiff, UK

**Keywords:** peripheral vision, size perception, shape perception, visual space, art

## Abstract

Little is known about how we perceive the size and shape of objects in far peripheral vision. Observations made during an artistic study of visual space suggest that objects appear smaller and compressed in the periphery compared with central vision. To test this, we conducted three experiments. In Experiment 1, we asked participants to draw how a set of peripheral discs appeared when viewed peripherally without time or eye movement constraints. In Experiment 2, we used the method of constant stimuli to measure when a briefly presented peripheral stimulus appeared bigger or smaller compared with a central fixated one. In Experiment 3, we measured how accurate participants were in discriminating shapes presented briefly in the periphery. In Experiment 1, the peripheral discs were reported as appearing significantly smaller than the central disc, and as having an elliptical or polygonal contour. In Experiment 2, participants judged the size of peripheral discs as being significantly smaller when compared with the central disc across most of the peripheral field, and in Experiment 3, participants were quite accurate in reporting the shape of the peripheral object, except in the far periphery. Our results show that objects in the visual periphery are perceived as diminished in size when presented for long and brief exposures, suggesting diminution is an intrinsic feature of the structure of the visual space. Shape distortions, however, are reported only with longer exposures.

## Introduction

Visual space is the subjective appearance of physical space (Hershenson, 1999). It can be distinguished from the visual field, which is the entire region of the world visible to both eyes during any one fixation (Gibson, 1950; Howard & Rogers, 1995). There is widespread agreement that visual space does not correspond faithfully to physical space (Foley, Ribeiro-Filho, & Da Silva, 2004; Hatfield, 2003; Indow, 2004; Koenderink & van Doorn, 2008; Ogle, 1950/1964; Wagner, 2006). But the precise ways in which physical space, the visual field, and visual space interact are still not fully understood.

This study addresses the structure of visual space, and in particular the perceived size and shape of objects when viewed in the peripheral visual field. Intuitively we might suppose that a disc viewed directly would appear just as big and just as circular when perceived in the periphery. But several studies report conflicting results (Bedell & Johnson, 1984; Collier, 1931; Drum, 1977; Grindley, 1931; Helmholtz, 1865; James, 1890; Newsome, 1972; Schneider, Ehrlich, Stein, Flaum, & Mangel, 1978; Stevens, 1908; Thompson & Fowler, 1980; Zigler, Cook, Miller, & Wemple, 1930). In an early case, Stevens (1908) found that discs viewed peripherally appeared larger than when viewed in the central region. However, Newsome (1972) obtained the opposite result when he asked participants to adjust the size of a stimulus viewed in the periphery by moving it closer or farther away until it matched that of a reference stimulus viewed centrally. He concluded that objects observed peripherally appear smaller than they do centrally, an effect that increases with eccentricity. He did, however, qualify his results because his experimental setup did not allow him to control for errant eye movements or the perceived distance of the stimuli from the viewer. Schneider et al. (1978) also reported a diminution of perceived object size in the periphery but observed the effect along both horizontal and vertical axes of the visual field. Thompson and Fowler (1980) obtained similar results. Bedell and Johnson (1984) found that luminance could alter perceived size of objects in the periphery, with more brighly lit objects tending to be overestimated in size and dimly lit ones underestimated. It seems, therefore, that the degree of eccentricity and level of luminance can affect the perceived size of objects in the periphery. We are not aware of any similar studies on the perceived shape of peripherally viewed objects.

**Figure 1.**
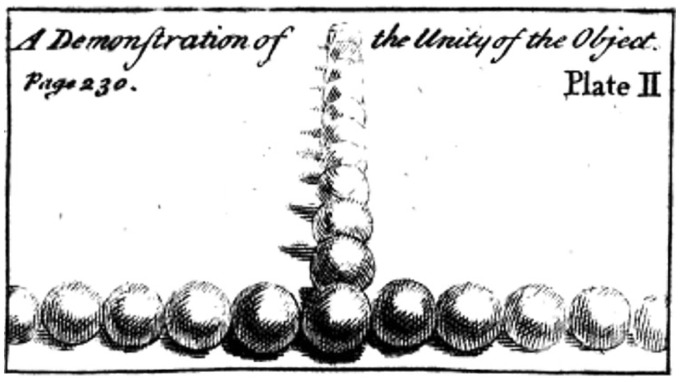
An illustration of the perceived diminution of objects in peripheral vision, taken from an 18th century artists’ textbook (De Piles, 1708). Note the diminution of the balls in the left and right periphery, which increases with eccentricity.

Questions about how objects are perceived across the visual field are also important to artists wishing to depict what they see. Artists have long been aware that the appearance of objects changes depending on where and how they are viewed (Du Fresnoy, 1695). The engraver and art critic Roger de Piles noted in his Principles of Painting that “Bodies decrease in both force and colour in proportion as they recede from the straight line, which is the centre of vision” (de Piles, 1708, p. 67). De Piles argued that paintings achieve compositional unity when the pictorial space is organized around a single point of focus. He illustrated this principle in the engraving shown in Figure 1. The balls receding into the distance and in the periphery become increasingly reduced in size, clarity, and contrast compared with the central fixated one.

**Figure 2.**
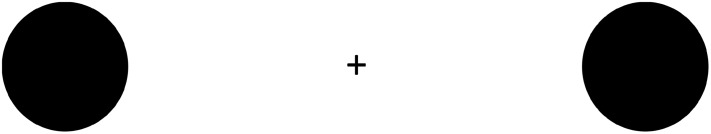
An apparent change in shape and size of peripherally viewed discs. Lining up the center point between the eyes with the cross, fixate on the center of either disc but then pay attention to the other, and then do the reverse. After approximately 3 seconds, you may notice the disc in your periphery appears significantly smaller and may even alter its shape. The apparent diminution occurs in both monocular and binocular viewing.

Several of our own observations about the structure of visual space correspond to the suggestions of de Piles, and some of the psychological literature cited earlier. The first observation was made during a project in which the aim was to make paintings and drawings that captured the full scope of visual experience associated with a given fixation point in space, including the entire peripheral field on a two-dimensional surface (Pepperell, 2012). These depictions differed in a significant and consistent way from linear perspective depictions of the same scenes. In particular, objects in the visual periphery appeared smaller and more compressed compared with those seen centrally. Objects in the horizontal axis appeared to be compressed in width, while objects in the vertical axis appeared to be compressed in height. The second was the finding that the same tendency was evident in the work of other artists, such as Paul Cézanne, Vincent van Gogh, and Canaletto (Mather, 2015; Pepperell & Haertel, 2014). Third was the finding that images generated according the principles described earlier were judged to more accurately depict a given scene than geometrical perspective depictions of the same scene (Baldwin, Burleigh, & Pepperell, 2014; Koenderink, van Doorn, Pinna, & Pepperell, 2016). Finally, we observed apparent size diminution and shape distortion in the peripheral field when swapping fixation between two identical objects, such as a pair of discs. We noted that after approximately 3 seconds of fixation the disc in the periphery appeared smaller and more elliptical in shape (see Figure 2). Perceiving this apparent size and shape distortion requires effort of a kind familiar to artists when “shifting experience away from the familiarity of ideas and toward the concrete immediacy of sensory perception,” as it is put in one widely used artists’ textbook (Curtis, 2002, p. 32). In psychological terms, this is the equivalent of dissociating the proximal stimulus from the perceived structure of the physical object. A recent study by Erkelens (2015) showed that participants were able to do this to a surprising extent when comparing judgments about the perceived length of railway tracks viewed in perspective pictures and in reality. They were able to report the apparent length of the tracks due to perspectival information and the physical length of the lines quite independently, even when the magnitiude of difference between them was very large.

The evidence from the psychological studies cited earlier and our observations from research in the visual arts led us to hypothesize that objects perceived in the visual periphery can appear smaller than identical objects seen in central vision, even when the perceiver knows they are the identical. In addition, we hypothesized that objects can appear compressed in the visual periphery compared with when seen in the center, with objects in the horizontal axis being compressed in width, and objects in the vertical axis being compressed in height (Pepperell, 2012). Again, this apparent compression can be perceived despite the knowledge that the objects are identical. The aim of this study was to test whether participants would report these apparent changes in size and shape when asked to judge the appearance of identical stimuli in different parts of the visual field and under different viewing conditions. If so, it could allow us to more clearly understand the differences between visual and physical space and also help to explain why artists have often recorded the appearance of the visual world using these principles.

## Experiment 1

Most previous studies comparing perception in central and peripheral vision have focused either on a relatively narrow region of the visual field, on the horizontal axis only, or on perceived changes in the size of objects rather than their shape (e.g., Bedell & Johnson, 1984; Masin, 2008; Newsome, 1972; Schneider et al., 1978; Tsal & Shalev, 1996). As artists are generally interested in recording the appearance of visual space across a wide angle of view in both axes, and the perceived shape as well as size of objects, our first experimental design accommodated all these aspects. In Experiment 1, our purpose was to investigate whether participants would report the apparent diminution and compression we had previously observed in artists when focusing on a point in space and drawing the contents of their visual periphery. Drawing is commonly used by artists to record visual experience. But it is also a well-established method of measuring subjective judgments in psychological experiments (Cohen, 2005; Cohen & Bennett, 1997; Mitchell, Ropar, Ackroyd, & Rajendran, 2005; Perdreau & Cavanagh, 2014).

### Method

#### Participants

Thirty-two undergraduate and postgraduate students (mean age of 21) from a variety of disciplinary backgrounds took part in the experiment. Twenty-four had normal vision, and eight had corrected-to-normal vision. Participants gave informed consent and were naïve about the purpose of the experiment. The experiment received the approval of the Ethics Committee of Cardiff Metropolitan University and was conducted in accordance with the Declaration of Helsinki (2008). Each participant received a £5 cafeteria voucher for taking part to the experiment.

#### Materials

**Figure 3.**
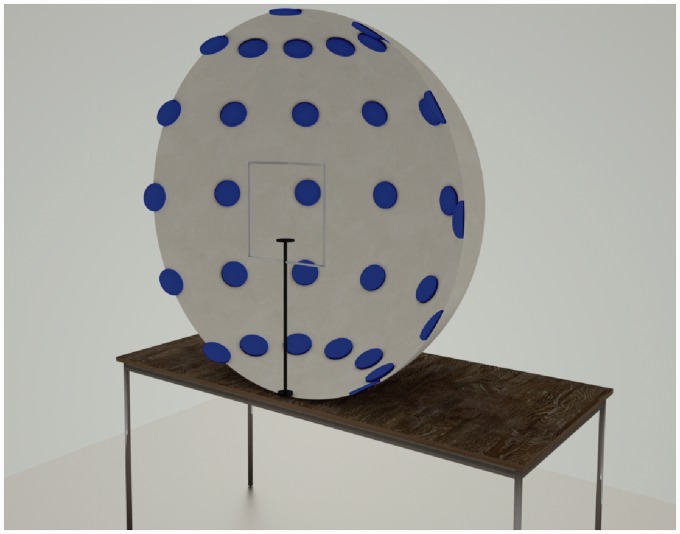
Illustration of the hemispherical dome apparatus used in Experiment 1. It shows the arrangement of the discs and the chin and headrest.

The experimental apparatus was the same used in a previous study by Baldwin et al. (2014). It consisted of a concave hemispherical dome of 900 mm diameter onto the surface of which were fixed 37 discs of 75 mm diameter. We arranged the discs at increments of 30° from the center, both along the horizontal and vertical axis (Figure 3). In this way we ensured the stimuli fell comfortably within the binocular visual field (Howard & Rogers, 1995). Participants were seated with their eyes 45 cm from the center of the dome, perpendicular to the central disc. In this position each participant’s visual field was fully encompassed by the apparatus. Participants’ heads were constrained by a forehead and a chin rest to ensure consistency of position relative to the center of the dome. An adjustable chair ensured participants’ eyes were at a uniform height.

The background surface of the dome was white, and the discs were blue. An indirect tungsten lamp evenly illuminated the scene and cast no shadows inside the dome. We used a SPER 840020 light meter to measure the luminance of the apparatus, and the constrast between discs and background. The discs had a Weber contrast value against the background of approximately −0.4% (luminance value of blue discs 2.52 cd/m^2^ and background 4.14 cd/m^2^).

#### Procedure

Participants who wore glasses were asked to remove them before starting the experiment to prevent the rims occluding their peripheral field. Once seated in the apparatus, participants were given a brief training session guided by the experimenter using a written protocol. The experimenter instructed participants how to pay overt attention to objects in the visual periphery while fixating on a central point in the apparatus. The aim of the training was to ensure participants fully understood the experimental task.

During the experiment, participants had to fixate on the central disc and pay attention to one of four peripheral discs as indicated by the experimenter. They then had to fixate on the selected peripheral disc while paying attention to the central disc. In each case they were asked to make a mental note of how the peripherally viewed disc appeared compared with the fixated one. The four peripheral discs were located (a) at 30° above and (b) 30° below the central disc, with their central points vertically aligned with the central disc, and (c) at 30° to the left and (d) 30° to the right of the central disc, with their central points horizontally aligned with the central disc. After viewing each of the four peripheral discs, participants had to draw the appearance of the peripheral disc compared with the central one. Participants were provided with four sheets of paper (420 × 420 mm) each with a blue disc of 75 mm diameter printed in the center. It was explained that the printed disc represented the central disc in the apparatus and was to be used as a reference for the drawings of the peripheral discs. Participants were allowed to look between the discs as many times as they wished and were allowed as much time as they needed to complete the drawings in order to be satisfied they had accurately represented what they perceived. The average time to complete the task was 15 minutes.

Each drawing was scanned and imported to Adobe Illustrator. Using a vector drawing tool, we placed a rectangular bounding box around the edge of each drawn disc and obtained a measure of the height and the width for each drawing of the peripherally viewed discs.

### Results and Discussion

**Figure 4.**
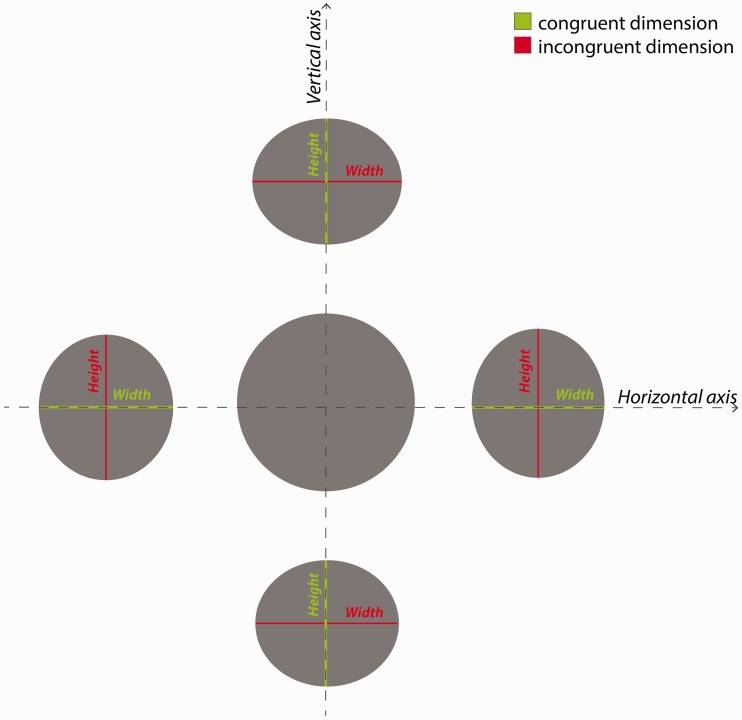
A graphical representation of the perceived size of the discs viewed peripherally. The four peripheral discs’ dimensions have been modified to reflect their perceived size based on the mean height and width values calculated from participants’ drawings in Experiment 1. The above and below discs’ height and the left and right discs’ width are the congruent dimensions to the aligned axis, represented in green. The above and below discs’ width and the left and right discs’ height are the incongruent dimensions to the aligned axis, represented in red. The horizontal and vertical axes are represented by dashed lines.

We first determined that there was no significant difference in the results between participants with or without corrected vision (see Supplemental materials, Experiment 1 for details). An initial qualitative analysis revealed two main characteristics of the drawings. The first was an overall diminution effect in which the drawn discs were smaller than the physical discs. The second was a shape or orientation effect in which the discs above and below the central disc were represented as horizontally oriented ellipses, being compressed in height compared with width, while the left and the right discs were represented as vertically oriented ellipses, being compressed in width compared with height. Figure 4 is a graphical representation of the four peripheral discs’ dimensions modified according to the mean height and width derived from the drawings in reference to the central disc.

To quantify the diminution effect, we calculated the bias for the drawn discs compared with the physical discs (Walther & Moore, 2005). If the drawn discs were reported as having the same size as the physical discs, then the expected value for the bias = 0. To quantify the shape or orientation effect, we calculated the aspect ratio of the drawn discs (AR = drawn disc’s width/drawn disc’s height). If the drawn discs were drawn as perfect circles (width = height), the expected value for the AR = 1.

A negative bias was found for all the dimensions of the drawn discs: above disc’s width =−.16, above disc’s height = −29, below disc’s width = −.19, below disc’s height = −.29, left disc’s width = .24, left disc’s height = −.18, right disc’s width = −.25, right disc’s height =−.16 (see Table 1 for more details). A single sample *t* test was conducted to determine if there was a statistically significant difference between the dimensions of the drawn discs and the physical discs in the apparatus (diameter = 75 mm). The dimensions of the drawn discs were significantly smaller (above width *M* = 63.2, *SD* = 15.13; above height *M* = 53.22, *SD* = 12.9; below width *M* = 60.64, *SD* = 14.5; below height *M* = 53.3, *SD* = 10.47; left width *M* = 56.7, *SD* = 2.1; left height *M* = 61.43, *SD* = 12.33; right width *M* = 56.06, *SD* = 12.2; right height *M* = 62.9, *SD* = 13.8) than the physical discs in the apparatus (above width *t*(31) = −4.42, *p* < .001; above height *t*(31) = −9.55, *p* < .001; below width *t*(31) = −5.6, *p* < .001; below height *t*(31) = −11.73, *p* < .001; left width *t*(31) = −8.6, *p* < .001; left height *t*(31) = −6.22, *p* < .001; right width *t*(31) = −8.76, *p* < .001; right height *t*(31) = −4.92, *p* < .001).

**Table 1.** Average Bias Values for Each of the Drawn Discs, Standard Deviation (SD), and Relative Root Mean Square Error (RRMSE) Calculated for the Drawn Discs’ Parameters (Width and Height) in Each Displayed Position Compared With the Value of the Physical Discs in the Apparatus (Diameter = 75 mm).

	Above width of drawn discs	Above height of drawn discs	Below width of drawn discs	Below height of drawn discs
Bias	−0.16	−0.29	−0.19	−0.29
*SD*	15.14	12.90	14.49	10.47
RRMSE	3.51	3.03	3.46	1.99
	Left width of drawn discs	Left height of drawn discs	Right width of drawn discs	Right height of drawn discs
Bias	−0.24	−0.18	−0.25	−0.16
*SD*	12.04	12.32	12.22	13.82
RRMSE	2.47	2.40	2.59	2.97

RRMSE = Relative Root Mean Square Error.

The results from the AR calculation showed that for the above and below discs the AR was >1 (average for above AR = 1.26; average for below AR = 1.16), meaning that the vertically aligned discs were consistently drawn with their width larger than their height; while for the left and right discs the AR was <1 (average for left AR = 0.94; average for right AR = 0.91), meaning that the horizontally aligned discs were consistently drawn with their height larger than their width.

**Figure 5.**
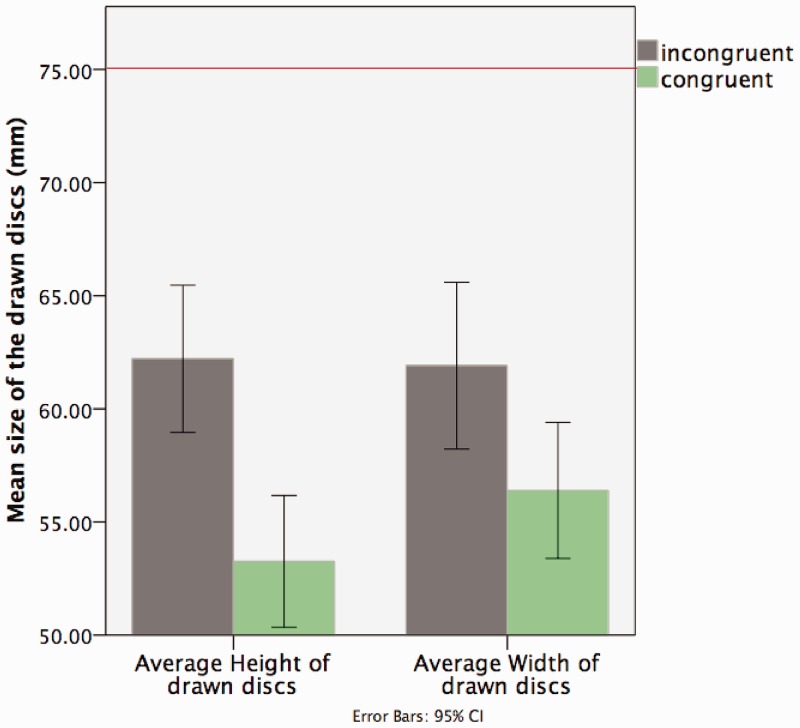
This graph shows the mean dimensions (mm) of the drawn discs (vertical axis of the graph) and the average height and width as measured from the drawings (horizontal axis of the graph) as a function of the congruent and incongruent conditions. The green bars show the average dimensions congruent to the alignment axis, while the blue bars show the average dimensions incongruent to the alignment axis. The congruent dimension for the vertically aligned discs (above and below) is the height and the incongruent dimension is the width, while the congruent dimension for the horizontally aligned discs (left and right) is the width and the incongruent dimension is the height. The red line indicates the dimension of the physical discs in the apparatus (diameter = 75 mm).

**Figure 6.**
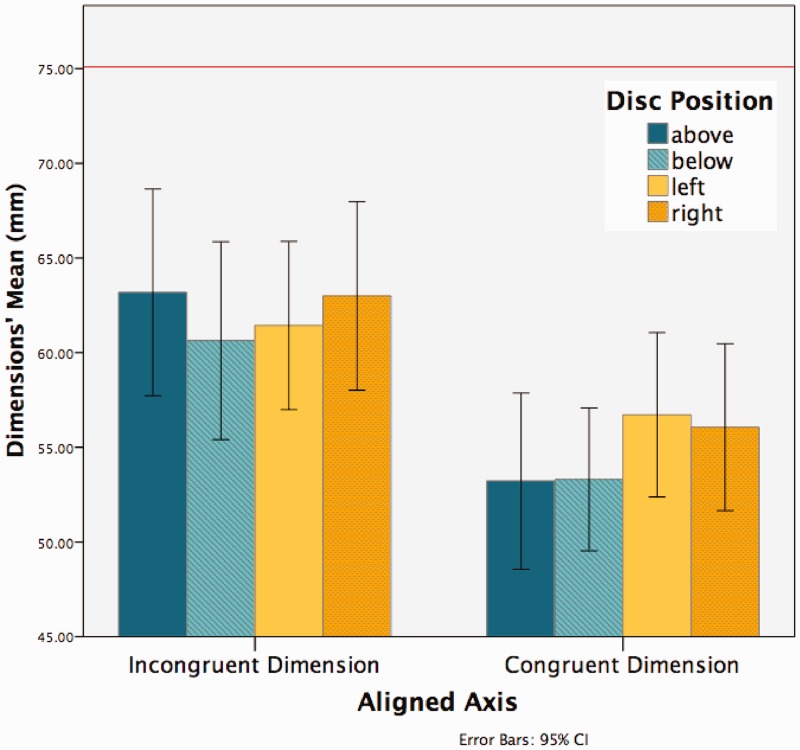
The graph shows for each position (abobe, below, left, and right) the average drawn discs’ dimensions (mm) as a function of the congruent and incongruent aligned axis. The incongruent dimension for above or below discs is the width and for the left or right discs it is the height; the congruent dimension for the above or below discs is the height and for the left or right discs is the width. The red line indicates the dimension of the physical discs in the apparatus = 75 mm.

Based on our previous qualitative analysis, we predicted that there would be a significant difference between the amount of compression in the height and width of discs depending on whether they were congruent or incongruent to the alignment axes (see Figure 4). To test this hypothesis, we conducted a 2 × 4 repeated measures analysis of variance (ANOVA; alignment axis: congruent dimension vs. incongruent dimension; disc position: above vs. below vs. left vs. right) on the drawn discs’ dimensions. We found a significant main effect of the alignment axis: *F*(1, 31) = 11.714, *p* < .005, partial η^2 ^= .274, meaning that the drawn discs’ dimensions congruent to the alignment axis were significantly smaller compared with the incongruent ones. The main effect of the disc position was not significant: *F*(1, 31) =1.416, *p* > .1, partial η^2 ^= .044. There was no significant interaction between the two factors: *F*(1, 31) = 2.516, *p* > .05, partial η^2 ^= .075. Results from the ANOVA confirmed that the drawn discs reported a statistically significant shape or orientation effect in the dimension congruent to their alignment axis compared with the incongruent one (see Figures 5 and 6).

**Figure 7.**
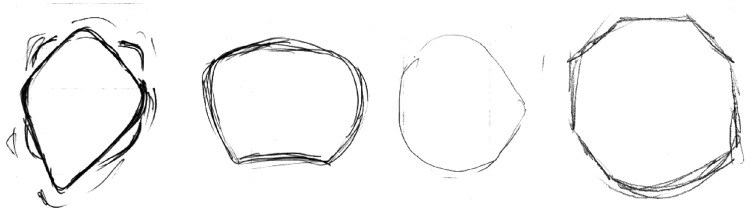
Examples of drawings from four different participants showing the perceived shape of objects seen in the peripheral field, with discs appearing polygonal in shape.

In addition to the changes in size and shape of the drawn discs, we report an effect in which 8 of the 32 participants explicitly drew the peripheral discs with polygonal contours. A similar effect has been reported previously in both directly perceived shapes (Khuu, McGraw, & Badcock, 2002; Sakurai & Beaudot, 2015) and afterimages of circular shapes (Ito, 2012). Figure 7 shows a sample of some polygonal drawings. Overall the results of Experiment 1 show that peripherally viewed discs appeared smaller and less circular than viewed centrally.

## Experiment 2

In Experiment 1 we allowed participants to move their eyes at will and to look at the stimuli for as long as they wanted, which resulted in reports of distortions in the perceived size and shape of peripheral discs. To investigate whether shape and size distortions in the periphery occurred independently and under more controlled conditions, we designed two further experiments. In Experiment 2, the diminution effect was studied. Stimuli of different sizes were presented briefly (200 ms) at different eccentricities with eye movements constrained using an eye tracker. We used a computer-controlled video projection on a curved screen to present stimuli at an equal distance from the participants’ eyes up to 60 horizontal degrees of binocular visual field in each hemifield. With this arrangement we were able to control for the possible influence of eye movements, stimuli distance, and exposure duration, all factors that Newsome (1972) had been unable to control for in his classic study. Based on the results of Experiment 1 and Newsome’s study, we predicted that the peripherally presented stimuli would be judged as smaller compared with a central reference disc and that this diminution would increase with eccentricity.

### Method

#### Participants

Seventeen participants (7 women, 10 men; mean age = 35, range 23–55) gave informed consent before taking part to the experiment. All were recruited from the student and staff population of Cardiff Metropolitan University. Twelve had normal vision, and five had corrected-to-normal vision. Participants who wore glasses removed them before starting the experiment to prevent the rims occluding their peripheral field. The experiment was approved by the Ethics Committee of the School of Art and Design, Cardiff Metropolitan University and was conducted in accordance with the Declaration of Helsinki (revised 2008). Each participant received a £5 cafeteria voucher for participating.

#### Materials

**Figure 8.**
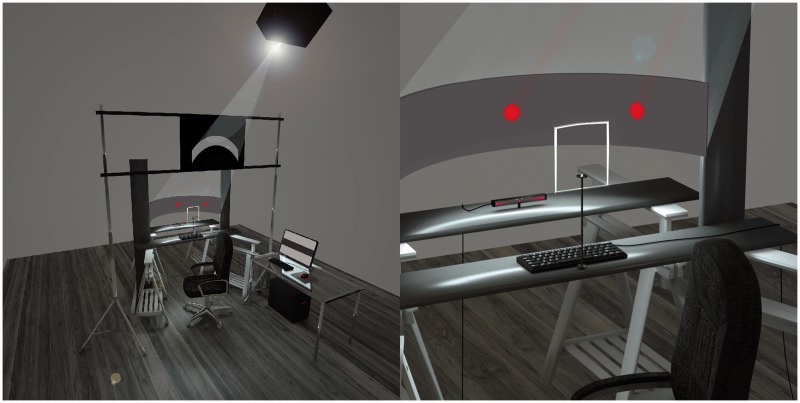
Illustration of curved screen apparatus used in Experiment 2. The left image shows the entire apparatus, including the control computer in the table to the right, and the right image shows in more detail the position of the screen, eye tracker, head restraint, and keyboard used for recording participants’ responses.

The apparatus consisted of a curved screen 22 cm high and 113 cm wide on which stimuli were presented (see Figure 8). The screen covered 120° of visual field, approximately corresponding to the area of the human binocular vision that accounts for the majority of the approximately 180° of the total human visual field (Gibson, 1950; Howard & Rogers, 1995; Strasburger, Rentschler, & Jüttner, 2011). We wanted to avoid using a flat computer monitor to present the stimuli because they make it difficult to maintain consistency in size and shape of the stimuli projected on the retina, especially at eccentricities of 40° or more (Yu & Rosa, 2010). The technical problems involved in presenting computer-controlled stimuli to a wide angle of the visual field may partly explain why researchers to date have tended to limit studies of peripheral vision to a relatively narrow range of eccentricities.

To ensure the curvature of the screen was constant across all the surface, we used a semicircular Computer Numeric Control (CNC) machine cut frame that had a diameter of 120 cm . An InFocus IN3128HD (60 Hz) projector, fixed on the roof of the lab, was used to project stimuli onto the screen. A mask layer was inserted between the projector’s light source and the screen itself to eliminate any light spillage around the screen in order to avoid any source of distraction (see Figure 8). The experiment was created using Python and PsychoPy (Peirce, 2007). We gamma corrected the screen using the default PsychoPy function. We used a SPER 840020 light meter to measure the background and the stimuli luminance values at each eccentricity taking into account the curvature of the screen. Then we adjusted each discs’ luminance to maintain a Weber contrast value against the background of −0.1% across the screen surface (background luminance: .062 cd/m^2^; average stimuli luminance: .057 cd/m^2^). This setting was used in order to minimize the formation of afterimages due to the luminance of the projection.

We created a set of stimuli that varied from half to double size of a central reference disc. Stimuli consisted of a series of red discs of nine fixed sizes varying from 0.75 cm to 3 cm, that is, from 50% to 200% of the size of a 1.5 cm central reference disc subtending 1.43° of visual angle. The shapes were generated in Adobe Illustrator and then laser cut into physical templates that were used to map the final projected digital stimuli at the correct size and shape on the curved screen using Adobe After Effects. Our apparatus was fitted with an Eye Tribe eye tracker (www.theeyetribe.com, Copenhagen) with a temporal resolution of 60 Hz. The eye tracker was used to detect if the participants’ eyes moved from the central fixation point, in which case the stimuli were blanked. A high quality 5 m long HDMI cable was used to link all display devices and minimize any computer to display lag. The experiment was coded in PsychoPy at a rate of 60 frames per second.

#### Procedure

**Figure 9.**
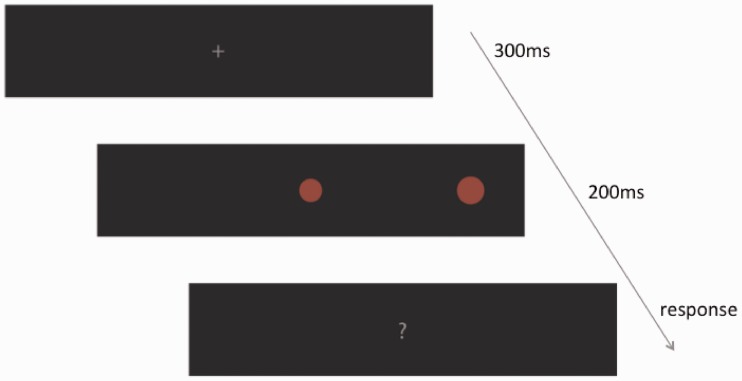
A graphical illustration of the sequence of events for each trial. First a fixation cross was shown for 300 ms, which the participants had to fixate on for the trial to be valid. Then a central reference disc (appearing at the same positions as the fixation cross) and a peripheral disc were presented for 200 ms. Finally a question mark indicated that participants had to judge whether the peripheral disc was smaller or larger than the central one by pressing a key.

Participants were seated in a darkened room at 60 cm from the surface of the screen. All external light sources were removed. We provided an adjustable chair to line up the participants’ eyes with the central fixation point on the screen. Participants’ heads were constrained by a forehead and a chin rest to ensure they were all located in the same position relative to the screen (see Figure 8). Before starting the experiment, participants were given a training session to test whether they were able to perceive the stimuli at all eccentricities and whether they understood the task. During the experimental session, each trial consisted of the following sequence of events (see Figure 9): first a fixation cross appeared at the center of the screen for 300 ms (18 Hz); then the stimuli appeared for 200 ms (12 Hz), one in the center of the screen and one in the periphery; then a question mark was shown. Participants performed a forced-choice size discrimination task in which they reported whether the peripherally viewed disc appeared larger or smaller than the centrally fixated disc. For half the participants “L” was used on a keyboard to report the disc appeared larger and “A” for smaller, and this was reversed for the other half of the participants.

We presented the stimuli using the method of constant stimuli. The peripheral stimuli were of the following sizes: 0.75 cm, 0.9 cm, 1.2 cm, 1.35 cm, 1.5 cm, 1.65 cm, 1.8 cm, 2.4 cm, and 3 cm. Each size was randomly presented 10 times at 15°, 30°, 45°, and 60° of eccentricity from the central fixation point in both hemifields (left and right).

### Results and Discussion

**Figure 10.**
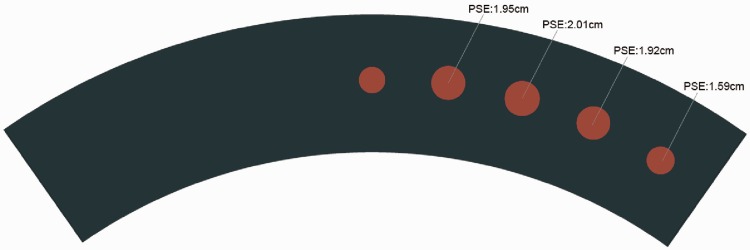
A graphical representation of the mean points of subjective equality at each eccentricity. The diameter of peripheral discs has been modified to reflect the mean points of subjective equality values calculated in Experiment 2. PSE = Points of Subjective Equality.

**Figure 11.**
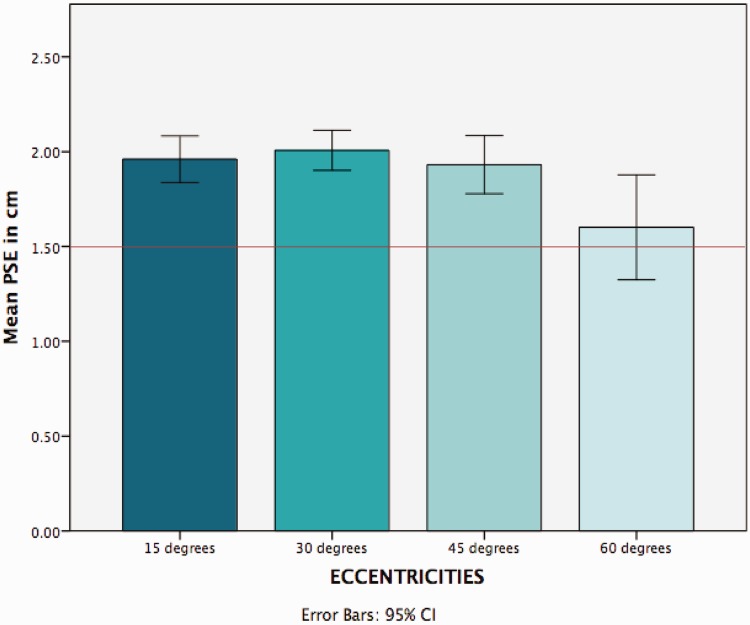
The graph shows the mean points of subjective equality values (mm) for each eccentricity (15°, 30°, 45°, and 60°) as calculated in Experiment 2. The red line indicates the physical size of the central reference disc. PSE = Points of Subjective Equality.

We excluded from the data analysis trials in which participants were not looking directly at the central disc and where response time was less than 250 ms or greater than 3000 ms. We performed a probit analysis to calculate the psychometrical function for each participant. The mean Points of Subjective Equality (PSE) for each eccentricity was: 1.95 cm at 15° eccentricity (130% of the central disc), 2.01 cm at 30° (134% of the central disc), 1.92 cm at 45° (128% of the central disc), and 1.59 at 60° (106% of the central disc; Figures 10 and 11).

Having established there was no significant difference between the PSE reported by participants with or without corrected vision (see Supplemental Materials, Experiment 2 for details), a one-way ANOVA within subjects was conducted on the PSEs at the four different eccentricities. There was a statistically significant effect of eccentricity, accounting for a small portion of the variance: *F*(3, 48) = 13.798, *p* < .001, η^2 ^= .463. The mean PSE for the four eccentricities was set at 1.88 cm (125% of the central disc), meaning that overall people perceived peripheral discs 25% smaller compared with the size of the central reference disc (=1.5 cm). A Bonferroni post hoc test revealed significant differences between the mean value of the PSE at 60° and at the other locations (15°, 30°, 45°; *p* < .01 for all tests). No other comparisons were significant (all *p* > .05). Figure 10 is a graphical representation of the mean PSE for each eccentricity, indicated by modified size of peripheral discs. The PSE showed a positive bias for each eccentricity: PSE at 15° bias = .31, PSE at 30° bias = .34, PSE at 45° bias = .29, PSE at 60° bias = .07 (see Table 2 for more details).

**Table 2.** Bias, Variance (VAR), Standard Deviation (SD), and Relative Root Mean Square Error (RRMSE) Calculated on the PSE for Each Eccentricity.

	15°	30°	45°	60°
Bias	0.31	0.34	0.29	0.07
*SD*	0.80	0.69	0.99	1.79
RRMSE	2.11	2.13	2.09	1.91

RRMSE = Relative Root Mean Square Error.

In this experiment we found that people perceived briefly presented stimuli as smaller when presented in the peripheral at eccentricities of 15°, 30°, 45°, and 60° compared with central vision. However at 60° of eccentricity we found an unexpected result in which the mean PSE (=1.59 cm) was closer to the actual size of the central disc (=1.50 cm) compared with the other eccentricities. We had expected the diminution in perceived size to increase with eccentricity, as reported by Newsome (1972). One possible explanation is that participants may have referred to an a priori internal criterion in which objects perceived in the far periphery are assumed to be closer to their peripersonal space, and therefore appear larger (Caggiano, Fogassi, Rizzolatti, Thier, & Casile, 2009).

## Experiment 3

Experiment 3 was designed to investigate whether the shape or orientation effect reported in Experiment 1 could occur with brief exposure and without directly viewing objects presented in the periphery. We also considered the possibility that the diminution effect reported in Experiment 2 was due to perceived shape distortion. We reasoned that if the peripherally viewed discs were perceived as ellipses or polygons, as in Experiment 1, then this may have also reduced their overall apparent size. Based on the results of Experiment 1, we predicted participants in Experiment 3 would report a distortion of perceived object shape in the peripheral field. To test this we asked participants to select the perceived shape of peripherally viewed discs and octagons from a range of seven different directly viewed reference shapes.

### Method

#### Participants

Nine participants (7 women, 2 men; mean age=22, range 19–28) gave informed consent before taking part to the experiment . All were recruited from the student and staff population of Cardiff Metropolitan University. All had normal vision. The experiment had received approval by the Ethics Committee of the School of Art and Design, Cardiff Metropolitan University and was conducted in accordance with the Declaration of Helsinki (revised 2008). Each participant received a £5 cafeteria voucher for participating.

#### Materials

Experiment 3 was conducted using the same apparatus we used for Experiment 2 but with a different set of stimuli. Either discs or octagons were presented in the periphery. Octagons were chosen partly because several participants in Experiment 1 reported seeing this shape and also because they closely resemble a disc and are therefore hard to discriminate. We generated a series of reference shapes consisting of a disc, a vertically oriented ellipse, a horizontally oriented ellipse, an octagon, a hexagon, a pentagon, and a triangle (see Figure 11). The AR of the disc = 1; the AR of the vertically and horizontally oriented ellipses = 1.8; the AR of the octagon, the hexagon, and the pentagon = 1.25; the AR of the triangle = 1.6. We projection mapped all the shapes to the screen following the same procedure as in Experiment 2.

#### Procedure

Participants sat in a darkened room 60 cm from the screen. We provided an adjustable chair to line up the height of the viewer’s eyes with the central fixation point on the screen. Participants’ heads were constrained by a forehead and a chin rest fixed on the external border of the desk, thus ensuring they were all located in the same position relative to the screen.

The experiment was again created using Python and PsychoPy (Peirce, 2007) with a stimuli onset using rate of 60 frames per second. Before starting the experiment, participants were given a training session to ensure they perceived the discs at all eccentricities and understood the task.

We randomly presented a disc or an octagon in the periphery with the same nine sizes as Experiment 2 (0.75 cm, 0.9 cm, 1.2 cm, 1.35 cm, 1.5 cm, 1.65 cm, 1.8 cm, 2.4 cm, and 3 cm) 6 times at each position (3 times on the left and 3 times on the right). The eccentricities were the same as Experiment 2, that is, 15°, 30°, 45°, and 60°.

**Figure 12.**
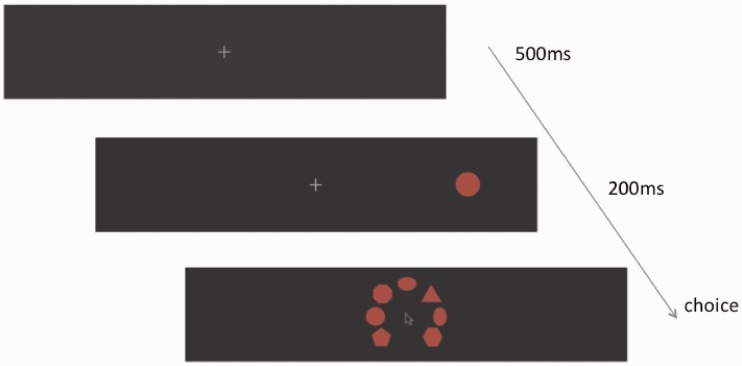
A graphical illustration of the series of events for each trial. First a fixation cross was shown for 500 ms, which the participants had to look at for the trial to be valid. Then a peripheral disc was presented for 200 ms. Then a set of shapes appeared in a random order on the screen with a cursor at the center of the screen. Participants had to click on the shape they thought was the closest to what they perceived in the periphery.

Each trial consisted in the following sequence of events (see Figure 12). First participants were presented with a fixation cross (500 ms). Then a peripheral stimulus (disc or octagon) was presented in the periphery at a random location (200 ms). After the stimulus was presented, the seven shapes appeared on the screen in a random order. A cursor appeared at the same screen location as the fixation cross at the same time as the seven shapes were displayed. We asked observers to click on which of the seven shapes was most similar to what they thought they perceived in the periphery.

We tracked participants’ eye movements with the Eye Tribe eye tracker (www.theeyetribe.com, Copenhagen) to ensure that participants were looking at the fixation cross at the center of the screen while making their judgments.

### Results and Discussion

We excluded trials from the data analysis in which participants were not looking directly at the central disc and where response time was less than 250 ms or greater than 3000 ms. For each participant we calculated the mean reaction times (Rts) at all eccentricities for both conditions in which discs or octagons were presented in the periphery (see Table 3). The results suggest that discs were easier to process (mean discs’ Rts = 1229 ms) compared with octagons (mean octagons’ Rts = 1274 ms). The percentage of correct responses when discs were shown in the periphery was 63%, while the percentage of correct responses when octagons where shown was 35.7%. Moreover, participants most frequently selected the shape that correctly matched the one presented in the periphery when the stimuli were at closer eccentricities (15° and 30°) compared with farther ones (45° and 60°). Here participants’ mean frequencies were more evenly distributed across all the shapes: 85.6% at 15°, 64.8% at 30°, 51.3% at 45°, and 46.6% at 60° when discs were presented; 50.4% at 15°, 36% at 30°, 30.6% at 45°, and 26% at 60° when octagons were presented. Contrary to what we expected, participants less frequently reported perceiving elliptical shapes compared with discs: 6.1% at 15°, 13.2% at 30°, 16.6% at 45°, 17% at 60°.

**Table 3.** Mean Reaction Times (ms) for Each Eccentricity (15°, 30°, 45°, and 60°) According to the Stimulus Shape Divided by Participants’ Response (Congruent vs. Incongruent).

	Disc shown		Octagon shown	
Eccentricities	Congruent (disc selected)	Incongruent (octagon selected)	Congruent (octagon selected)	Incongruent (disc selected)
15°	1241 ms	1217 ms	1299	1427 ms
30°	1264 ms	1244 ms	1304	1204 ms
45°	1166 ms	1157 ms	1254	1341 ms
60°	1200 ms	1342 ms	1255	1253 ms
Average	1229 ms	1269 ms	1274	1293 ms

We found that participants were more accurate in responding when discs where shown (discs’ *d*′ = 0.46) compared with octagons (octagons’ *d*′ = 0.46) and that also varied across eccentricities (see Table 4). To test if this was due to a differential sensitivity for the two shapes or to an effect of eccentricity, we calculated *d*′ for both discs and octagon at all eccentricities. A 2 × 4 ANOVA within subjects was conducted that examined the effect of presented shapes (discs vs. octagons) and eccentricity (15° vs. 30° vs. 45° vs. 60°) on the relative *d*′ values. There was no statistically significant interaction between the effects of presented shape and eccentricity on *d*′ values: *F*(3, 24) = .256, *p* > .05, η^2 ^= .031. Simple main effect analysis showed that there was no significant difference in sensitivity between discs and octagons, *F*(1, 8) = .001, *p* > .05, η^2 ^= .000, but that there was a significant main effect of eccentricity: *F*(3, 24) = 25.624, *p* < .001, η^2 ^= .762, meaning that people were significantly more accurate at closer eccentricities compared with farther ones.

**Table 4.** *d*′ Values for Both Shapes (Discs and Octagons) at Each Eccentricity (15°, 30°, 45°, and 60°).

	Discs *d*′	Octagons *d'*
15°	1.35	1.36
30°	0.56	0.61
45°	0.14	0.01
60°	−0.01	0.04
Average	0.46	0.42

*Note.* The last row (average) shows the mean *d*′ values for all eccentricities.

**Figure 13.**
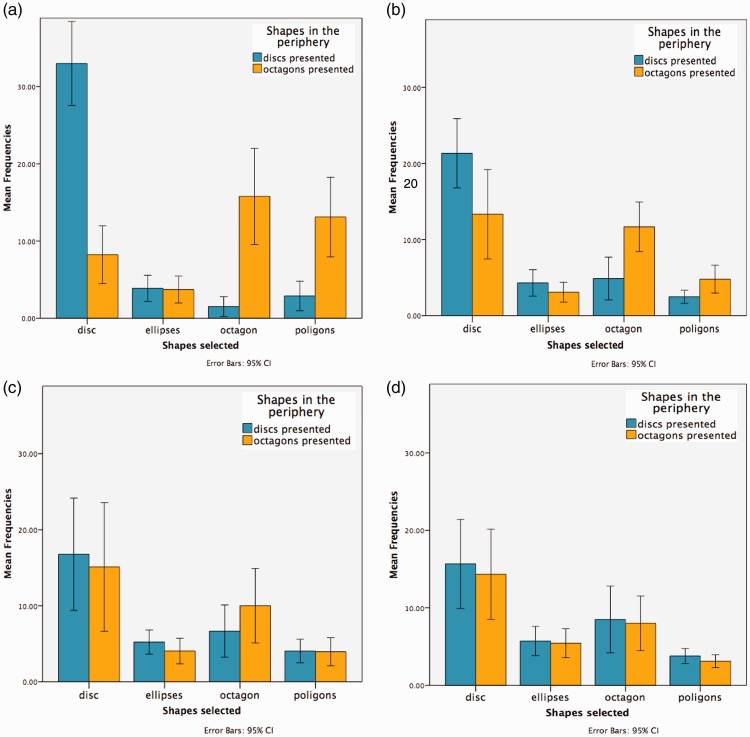
These graphs show the mean frequencies of selected shapes in the condition in which discs and octagons were presented in the periphery at different eccentricities. The blue bars show the mean frequencies when discs are presented peripherally, and the orange bars show the results when octagons were presented peripherally, as a function of the selected response category: disc, octagon, ellipses (vertically and horizontally oriented), and polygons (hexagon, pentagon, and triangle). (a) 15 degrees, (b) 30 degrees, (c) 45 degrees and (d) 60 degrees.

Our results suggest that up to 45° of eccentricity people can accurately discriminate between shapes even if they are briefly presented, showing faster Rts for and higher sensitivity to discs compared with octagons. At 45° and 60° of eccentricity, sensitivity values rapidly decreased close to chance level. Moreover at 60°, the mean *d*′ value for discs was negative, meaning that false alarms rates were higher than the correct response rates. This can be explained by the fact that we calculated the *d*′ only on the responses to discs. As eccentricity increased, participants’ mean frequencies were more evenly distributed across all the shapes, meaning that they perceived ellipses more often compared with closer eccentricities (see Figure 13) and this might have influenced our results. The fact that up to 45° participants were able to accurately perceive discs at brief presentations (200 ms) confirmed that the results we obtained in Experiment 2 were due to a diminution effect and not to a shape or orientation effect. The significant shape or orientation effect reported in Experiment 1 therefore seems to occur in the later stages of perception.

## General Discussion

In Experiment 1, we found that participants reported diminution and compression (shape or orientation effect) of objects perceived in the periphery without constraints on fixation or time. These results indicate that the size and shape of peripherally perceived objects can change in a way that is consistent with previous scientific studies and artistic observations. This effect occurred in spite of participants’ knowledge about the physical properties of the peripherally viewed objects. These findings could help to explain why artists have often represented visual space using similar principles of peripheral diminution and compression (Mather, 2015; Pepperell & Haertel, 2014). The picture changes somewhat when participants had to make judgments referring only to their peripheral vision under short time exposure. In Experiment 2, we still recorded the diminution effect in perceived size but did not find an equivalent shape-orientation effect in Experiment 3 to that reported in Experiment 1. It is important to underline that, unlike the first experiment, in Experiments 2 and 3 participants had no direct knowledge of size or shape of the presented stimuli and made judgments relying only on their peripheral vision. Overall our research suggests that in early stages of perception objects in the periphery are perceived as smaller than they appear in the central visual field, but that shape is perceived accurately up to 30° of eccentricity, approaching chance level at 45° and 60°. However under longer time exposures condition, discs were represented as ellipses, being compressed in height if aligned on the vertical axis and compressed in width if aligned on the vertical axis.

It is well known that the acuity of vision varies across the visual field, and that this can affect the way objects are perceived depending on their eccentricity (Helmholtz, 1867). Yet, due to the fact the region of space on which we fixate is seen with the highest acuity, we have the impression that all our visual field is uniformly clear and stable (Gibson, 1950). Artists, however, are trained to pay great attention to the way objects appear in visual space as a whole. Poussin, the great French Neoclassical painter, wrote: “There are two ways of looking at things. One is simply looking at them where the other is considering them attentively” (in Protter, 1997, p. 69). Paying greater attention to the contents of visual experience, which requires prolonged looking, is believed to heighten perceptual acuity and so enable greater representational accuracy. One popular training book for artists advises: “The more closely we pay attention to the information transmitted by the eye the more startled we will be” (Seth Jacobs, 2013, p. 29). This may account for the fact that artists have recorded the diminution and compression of peripherally viewed objects, while this phenomenon goes unnoticed by those not subjecting their visual experience to the same prolonged scrutiny. Understanding the strategies used by artists and other experts for widening the attention across the visual field is a promising direction for future research in visual perception (Hubert-Wallander, Green, & Bavelier, 2011; Hütterman, Memmert, & Simons, 2014).

Various proposals have been made to account for the differences in size perception of objects seen centrally and peripherally. Newsome (1972) cites the relative impoverishment of acuity in the peripheral field and structural properties of the eye as possible explanations, along with the depth distorting effects of the binocular horopter but concludes none of these satisfactorily account for his results. Bedell and Johnson (1984) suggest that a number of factors could influence peripherally perceived size, including the relative sensitivity of the retina between the fovea and periphery, the optical quality of the images projected onto the retina as well as the contrast and luminosity values of the target stimuli. They attributed their own results to the increase in receptive-field diameter with retinal eccentricity, which degrades the precision with which the stimulus is represented resulting in underestimated size. More recent work has attributed size perception to the cortical magnification factor of the foveal region compared with the periphery (Schwarzkopf, Song, & Rees, 2011). As a consequence of the distribution of retinal ganglion cells, there is an enlargement effect of foveal vision, such that identically sized objects seen peripherally will appear smaller compared with those seen centrally (Anstis, 1998). The results obtained using our novel curved screen apparatus confirmed that peripheral diminution effect occurs during both short (200 ms) and long (3–10 seconds) exposure times, and so extending previous findings up to 60° of eccentricity.

It is possible that the compression (shape-orientation effect) in peripheral field reported in drawings from Experiment 1 is caused in part to the way light is projected onto the retina through the cornea. Drasdo and Fowler (1974) used trigonometric ray tracing to calculate the projection of the retinal image, and showed the surface area onto which a solid degree of light is projected decreases markedly with eccentricity. In the 80° to 90° region of the retina the area covered is 37% of that in the foveal region. Due to the roughly spherical structure of the eye, this results in a pattern of optical distortion consistent with the observations reported in Experiment 1 in which objects appear increasingly horizontally compressed if aligned to the horizontal axis and vertically compressed if aligned to the vertical axis. However, on this basis we would expect the same compression effect to occur even with short exposures, and the results of Experiment 3 do not show this. One possible explanation is that perception of the retinal image is overridden by size constancy effects. Valsecchi and Gegenfurtner (2016) showed how the visual system maintains the appearance of a stable world even at early stages of perception by constantly recalibrating how objects appear when viewed centrally based on predictions about how they appear peripherally. Judgments about size in early perception, therefore, depend more on constantly recalibrated experience rather than on retinal images (Valsecchi & Gegenfurtner, 2016). Following this, we suggest that the longer exposure times in Experiment 1 may have led to greater awareness of the peripheral retinal image, which according to Drasdo and Fowler (1974) would be distorted, thus overriding the constancy effect and resulting in the shape compression reported in the drawings.

The appearance of polygonal shapes in the place of regular discs reported in Experiment 1 may also be due to prolonged exposures. Ito (2012) suggests that the appearance of curved lines as polygonal in afterimages perceived peripherally may result from rivalry between visual processes for detecting curves and corners in cortical areas. Adaptation or fatigue of one process may lead the other gaining dominance. In our first experiment, participants were able to peripherally view the discs for long periods, which may have resulted in adaptation or fatigue of the kind Ito describes. While Ito (2012) focused his study on the perceived shape of afterimages we have confirmed the same distortion occurs in the perception of physical stimuli, which suggests this may be a feature of visual perception more generally (Khuu et al., 2002; Sakurai & Beaudot, 2015). These findings may have interesting implications for the well-known aesthetic preferences for curvature (Bar & Neta, 2006; Gómez-Puerto, Munar, & Nadal, 2015; Leder, Tinio, & Bar, 2011) the effects of which have not yet been studied in the far periphery.

## Conclusion

Our study suggests that for longer time exposures objects in peripheral vision appear smaller and compressed in shape compared with central vision, and sometimes having polygonal contours. Objects aligned on the vertical axis appear compressed horizontally and objects aligned on the horizontal axis appear compressed vertically. These findings are consistent with several previous scientific studies and artistic observations. They further suggest that peripheral diminution and compression may be general features of the structure of visual space under certain viewing conditions, but further experimentation across the entire visual field need to be done in order to confirm this hypothesis. For brief time exposures, peripherally viewed stimuli also appear to be smaller than a central reference one but do not alter their perceived shape. That we are generally unaware of variations between the appearance of central and peripheral vision in everyday experience may be explained by tendency of the visual system to maintain a stable visual world through constancy effects. However, such constancy effects can be overridden when greater and longer attention is given to how objects appear in the peripheral visual field. In seeking to accurately depict their visual experience, artists may have recorded these size and shape variations in works of art when viewing their subject matter for prolonged periods, and this may account for the way those works are composed. These findings may contribute to our understanding of the structure of visual space and the ways in which artists have depicted visual experience.

## Author note

The author names are presented alphabetically in the author field.
